# Validating the Traditional Chinese version of the Epilepsy Anxiety Survey Instrument (EASI) in Hong Kong

**DOI:** 10.3389/fneur.2025.1604317

**Published:** 2025-07-08

**Authors:** Terry K. W. Chan, Mimi M. C. Wong

**Affiliations:** Department of Psychiatry, United Christian Hospital, Kowloon, Hong Kong SAR, China

**Keywords:** anxiety disorders, epilepsy, screening, epilepsy-specific anxiety, EASI

## Abstract

**Background:**

Anxiety disorders are common and have a substantial impact on people with epilepsy (PWE). However, they often go under-recognized. In recent years, epilepsy-specific anxiety has gained increasing attention in the literature. To improve mental health care for people with epilepsy (PWE), we developed and validated the traditional Chinese versions of the Epilepsy Anxiety Survey Instrument (EASI) and its brief screener (brEASI) among PWE in Hong Kong.

**Method:**

We developed the TC-EASI through forward and backward translation, followed by a review by an expert panel and a focus group. We tested the instrument among PWE aged 18 years and older using the Chinese Bilingual version of the Structured Clinical Interview for the Diagnostic and Statistical Manual of Mental Disorders, 4th Edition, Text Revision (CB-SCID), which is the gold standard for diagnosing anxiety disorders. We examined the internal consistency and the test-retest reliability of the TC-EASI and TC-brEASI. We performed confirmatory factor analysis (CFA) to assess the factor structure of the TC-EASI. We also examined convergent and divergent validity using the Generalized Anxiety Disorder 7-item scale (GAD-7), the Neurological Disorders Depression Inventory for Epilepsy (NDDI-E), the Depression Anxiety Stress Scales 21 (DASS-21), and the Liverpool Adverse Events Profile (LAEP). Furthermore, we analyzed the receiver operating characteristics (ROC) of the TC-brEASI.

**Results:**

We included 203 Chinese PWE; 19.7% had at least one current anxiety disorder, 7.4% had a panic disorder, and 5.9% had agoraphobia without panic disorder. Both internal consistency and test-retest reliability were satisfactory. The TC-EASI revealed two latent constructs: epilepsy-specific anxiety and typical anxiety. Convergent and divergent validity were established. A cut-off score of ≥9 for the TC-brEASI yielded a sensitivity of 89.2% (95% CI = 79.2–99.2%), a specificity of 82.5% (95% CI = 76.8–88.3%), and an area under curve (AUC) of 0.925 (95% CI = 0.887–0.964).

**Conclusion:**

The traditional Chinese versions of the EASI and brEASI are reliable and valid epilepsy-specific measures.

## 1 Introduction

### 1.1 Anxiety disorders in people with epilepsy

Epilepsy has a distinctive bidirectional interaction with anxiety disorders (AD). In addition to evidence on their linkage from biochemical (1, 2) and structural perspectives (3), research has also focused on the clinical and sociodemographic correlates of AD in people with epilepsy (PWE) (4–6), including the temporal occurrence of anxiety in relation to seizures. Anxiety can manifest as auras (7, 8), ictal fear and anxiety (9, 10), and postictal anxiety (11).

Scholars have recently attempted to delineate the atypical spectrum of interictal anxiety presenting in PWE (12–15). According to Hingray et al., the four main epilepsy-specific aspects of anxiety include anticipatory anxiety of epileptic seizures, seizure phobia, epileptic social phobia, and epileptic panic disorder.

Thus, AD in PWE does not appear to be a uniform and typical comorbidity of epilepsy but rather encompasses an atypical spectrum in the pathophysiological and clinical senses. Research on epilepsy-specific anxiety is still nascent, and more studies are needed to explore its concepts and implications. As such, instruments to better identify anxiety in PWE—including epilepsy-specific anxiety—would be pivotal; this would facilitate a better understanding of the psychiatric needs of PWE, improved management strategies, and, in the long run, enhanced mental health coverage for PWE.

### 1.2 The current situation for detecting anxiety disorders in people with epilepsy

A large population would benefit from improved detection of anxiety disorders associated with epilepsy, which is a common neurological disease both worldwide (16) and in Hong Kong (17). Comorbid AD is prevalent in PWE globally (18, 19) and also possibly in Hong Kong, as limited evidence suggests that anxiety is probable in more than 30% of adolescents with epilepsy (20) and 22 to 41% of adults with epilepsy (21). In a systematic review and meta-analysis, anxiety had an intermediate standard effect size on the quality of life (QoL) of PWE (22). Further, anxiety is the strongest predictor of their QoL, more so than seizure severity and frequency (23, 24), which are linked to worse medical outcomes (25, 26), higher healthcare costs (27), and an increased risk of suicide and premature mortality in PWE (28, 29).

Despite being prevalent and impactful, affective disorders in PWE remain under-recognized and improperly treated (30). A survey among epilepsy health professionals conducted by the International League Against Epilepsy (ILAE) in 2021 (31) revealed that over 90% of epilepsy health professionals agreed that the management of depression and anxiety is integral to epilepsy care, but only 40% felt adequately resourced. Two-fifths stated that they do not perform regular screenings for depression and anxiety, while only one-third said they use validated screening instruments; this is concerning because relying on unaided clinical judgment may cause AD to go underdiagnosed (32). The problem is also attributed to the patient factor. Utilization of mental health services by people with AD is suboptimal. People with AD exhibit physical symptoms, often in non-psychiatric settings (33, 34); this affects healthcare usage patterns (35) and increases the healthcare and economic burden (36), yet people with AD are underdiagnosed (37). A Hong Kong study indicated that < 50% of participants with AD had received mental health services in the past 12 months (38).

The international medical community is aware of this “diagnostic gap.” To achieve universal mental health coverage, the World Health Organization (WHO) launched an initiative to integrate mental healthcare into non-psychiatric settings (39). In 2011, the ILAE and, in 2015, the American Academy of Neurology advocated for psychiatric screenings using epilepsy-specific diagnostic tools (40, 41).

The abovementioned circumstances compellingly depict the dire need to develop and validate epilepsy-specific screening tools to explore and address the mental health needs of PWE in Hong Kong.

### 1.3 The Epilepsy Anxiety Survey Instrument (EASI)

In 2019, the EASI and its brief counterpart (brEASI) were developed as the first self-report, epilepsy-specific measures for anxiety (42). The 18-item EASI comprehensively assesses both the nature and severity of anxiety; it consists of two dimensions—“epilepsy-specific anxiety” and “typical anxiety”—that help clinicians determine whether anxiety is epilepsy-specific (e.g., excessive worry about seizures) or independent of epilepsy. It was also designed to avoid confounds based on epilepsy features or anti-seizure medications. Anxiety is portrayed in terms of neurological, psychological, and social factors, guiding patient-tailored management. The 8-item brEASI is a subscale derived from the EASI, designed to accurately detect all AD in the Diagnostic and Statistical Manual of Mental Disorders, Fifth Edition (DSM-5), among PWE.

The internal consistency of both measures was good (42). The authors identified a cut-off score of 7 for the brEASI, along with a sensitivity of 76%, specificity of 84%, and an area under the curve (AUC) of 0.891 (42). It outperformed the Generalized Anxiety Disorder 7-item scale (GAD-7) in detecting both GAD and non-GAD (42). The brEASI can thus be paired with the Neurological Disorders Depression Inventory for Epilepsy (NDDI-E) to establish a routine screening for PWE to improve the quality of psychiatric care for those with epilepsy.

Previously, the EASI was validated in French (43), German (44), Russian (45), and simplified Chinese in Western China (46), with satisfactory sensitivity (83.6–92.3%), specificity (72.6–92.5%), and AUC (0.828–0.916), but not in traditional Chinese or the local context of Hong Kong.

Although a simplified Chinese version had already been developed for Western China (46), several important reasons called for further validation. First, most written language in Hong Kong is traditional Chinese, and the majority of people speak Cantonese (47); these linguistic and cultural features contrast with the standard language of mainland China, which is simplified Chinese or Putonghua (48). Additionally, the study in Western China utilized a relatively small sample size (46). In addition, factor analysis of the EASI (which helps to assess construct validity) and test-retest reliability were not performed (46). As such, further validation is helpful to understand the EASI's psychometric properties and applicability in the context of Hong Kong.

### 1.4 Objectives

This cross-sectional study aimed to develop and validate the traditional Chinese versions of the EASI (TC-EASI) and its brief subscale (TC-brEASI) by examining their reliability and validity in a sample of Chinese adults with epilepsy at a specialized epilepsy outpatient clinic in Hong Kong. We posited that the measures would be valid and reliable tools for assessing anxiety symptoms among people with epilepsy (PWE) in Hong Kong.

## 2 Materials and methods

We obtained authorization from Dr. Amelia J. Scott and Prof. Louise Sharpe, the original authors of the EASI, to translate and validate its traditional Chinese version. Our study was approved by the Research Ethics Committee of the Kowloon Central/Kowloon East Cluster of the Hospital Authority of Hong Kong.

### 2.1 Development of the TC-EASI

The original English version of the EASI was translated into an initial draft in traditional Chinese by a bilingual psychiatrist and an independent translator.

An expert panel of nine members (including three senior psychiatrists, three specialist neurologists, two clinical psychologists, and one occupational therapist) was convened to evaluate its content validity. Modifications were made based on their feedback. A consensus was reached that the TC-EASI is relevant to and representative of local epilepsy patients with AD. The scale was then discussed in a focus group that included two patients with epilepsy and two patients with AD, who found the scale applicable and acceptable to local patients.

Back-translation into English was performed by another psychiatrist and another professional translator. Subsequently, the original authors of the EASI, the principal investigator of this study, the first translator, and a specialist psychiatrist compared the original English and the back-translated versions. The conceptual expression, cultural context, fluency, and clarity were considered. Amendments were made to the traditional Chinese version until the back-translated version was comparable to the original English version.

The TC-EASI was then pilot-tested on 10 epilepsy patients at the epilepsy clinic of the Department of Medicine and Geriatrics (M&G) at the United Christian Hospital (UCH) in Hong Kong.

The final versions of the TC-EASI and TC-brEASI are shown in the Supplementary material of this article. Responses are rated on a 4-point Likert scale, consisting of 0 (*not at all*), 1 (*several days*), 2 (*more than half the days*), and 3 (*nearly every day*). The TC-EASI score ranges from 0 to 54, while the TC-brEASI score ranges from 0 to 24.

### 2.2 Recruitment of participants

Participants were recruited from the specialized epilepsy clinic of the Department of M&G at UCH, which provides epilepsy outpatient services to a total of 648 adults in the area.

The sample size was estimated based on the requirements of confirmatory factor analysis (CFA) (49). A convenience sample of eligible patients was recruited starting from November 21st, 2022 until the sample size met the requirement. A list of all outpatients was retrieved from the clinical system and screened. The inclusion criteria were (1) being ethnically Chinese, (2) being 18 years or older, (3) being able to understand traditional Chinese and complete the questionnaire, and (4) having a neurologist-confirmed diagnosis of epilepsy according to the ILAE criteria (50). The diagnosis of epilepsy was documented clinically and verified, where necessary, with electroencephalograms. The exclusion criteria were (1) having a diagnosis of psychogenic non-epileptic seizures (PNES) or (2) having a diagnosis of intellectual disability or other neurological or psychiatric disorders that would prevent the participant from completing the questionnaire. The diagnosis of PNES had to be clinically established by a neurologist according to the ILAE criteria (51). Eligible patients who had visited the clinic during the recruitment period were invited to participate. The study was introduced, and informed consent was obtained.

**Figure 1:**
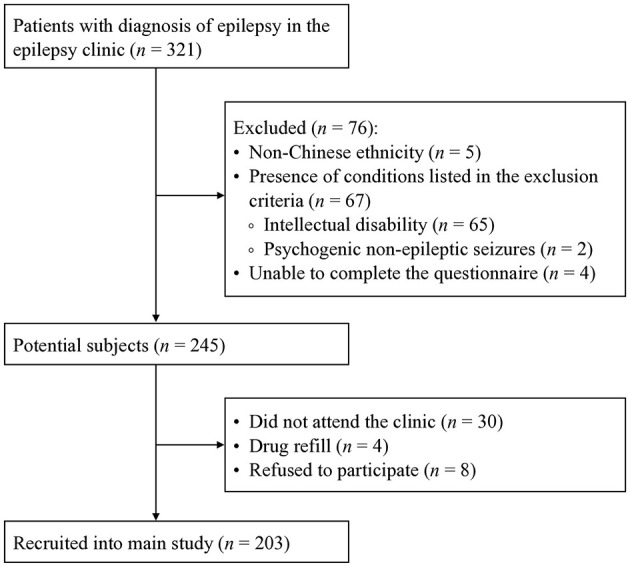
Recruitment flowchart.

Figure 1 shows the recruitment flow. Based on the selection criteria, a total of 76 patients were excluded, including five of non-Chinese ethnicity, 65 with intellectual disabilities, two with PNES, and four who were unable to complete the questionnaire. The remaining 245 patients were potential participants. A total of 30 of them had not visited the clinic, four were on drug refills, and eight refused to participate. Finally, 203 eligible patients participated in the main study. Recruitment ended on April 24th, 2023. The participation rate was 82.9%. As depicted in Table 1, the two groups did not differ significantly in terms of age or sex. Among them, 25 completed the TC-EASI again after 2 to 4 weeks.

**Table 1:** Comparison between participants and non-participants.

**Characteristics**	**Participants (*n* = 203)**	**Non-participants (*n* = 42)**	***p*-value**
Age, median (IQR)^a^	41 (31–57)	39 (26.75–57)	0.443
Sex, *n* (%)^b^			0.741
Male	91 (44.8%)	20 (47.6%)	
Female	112 (55.2%)	22 (52.4%)	

IQR, interquartile range.

^a^Mann-Whitney U test. ^b^Chi-square test. All *p* > 0.05.

### 2.3 Measurements

For each participant, socio-demographic information (age, sex, education level, employment status, and marital status), clinical information related to epilepsy (age of onset, duration of illness, type and focus, past-year seizure frequency, duration of seizure remission, and drug-resistant and resolved epilepsy), and treatment information (current anti-seizure medications, antidepressants, benzodiazepines, and vagal nerve stimulation) were collected.

Drug-resistant epilepsy is defined by the treating neurologist (according to the ILAE criteria) as the failure of adequate trials of two tolerated and appropriately chosen anti-epileptic drug regimens to achieve sustained seizure freedom (52). Epilepsy resolution is also established by the treating neurologist (according to the ILAE criteria) as either having passed the applicable age for age-dependent epilepsy syndrome or remaining seizure-free for the past 10 years while being off anti-seizure medications for at least the past 5 years (50). The duration of seizure remission refers to the number of months free from all types of seizures.

Additionally, the principal investigator administered the Chinese Bilingual version of the Structured Clinical Interview for DSM-IV-TR Axis I Disorders, Research Version, Patient Edition (CB-SCID-I/P) (53). To minimize assessment bias, it was performed before the collection of the self-report questionnaires, which included the TC-EASI, the Taiwanese NDDI-E (54), the Chinese version of the GAD-7 (55), the Chinese version of the Depression Anxiety Stress Scales 21 (DASS-21) (56), and the Chinese version of the Liverpool Adverse Events Profile (LAEP) (57). The self-report questionnaires were in paper-and-pencil format and completed in a private room at the epilepsy clinic, where participants could contact the investigator for questions as needed. For retesting, participants were randomly invited to complete the TC-EASI again in the clinic after 2–4 weeks. We obtained authorization from the authors of these assessments.

#### 2.3.1 The Chinese Bilingual version of the Structured Clinical Interview for DSM-IV-TR Axis I Disorder, Research Version, Patient Edition (CB-SCID-I/P)

The CB-SCID-I/P (53) is the Chinese version of the SCID-I/P, a semi-structured diagnostic interview used to determine DSM-IV-TR diagnoses (58). The inter-rater reliability, measured by percentage agreement for the principal diagnosis, was 87% for psychiatric patients in a multi-site study (59). The overall kappa value for AD was 0.81, while those for panic disorder, obsessive-compulsive disorder (OCD), and GAD were 0.80, 0.77, and 0.77, respectively (60). The principal investigator received training in conducting the CB-SCID-I/P prior to the study and achieved an intra-class correlation of over 0.8 for inter-rater reliability with a qualified psychiatrist at the same hospital. The full interview, including major modules to diagnose mental disorders, was administered and required around 40–70 min each time.

In the CB-SCID-I/P, where DSM-IV-TR diagnoses are made, OCD and post-traumatic stress disorder (PTSD) are classified as AD (Module F). However, in DSM-5, they are placed in different chapters. In this study, which utilized the CB-SCID-I/P, we included OCD and PTSD in AD. We conducted a subgroup analysis to identify any significant differences in the TC-EASI scores after excluding participants with OCD and PTSD.

#### 2.3.2 The Taiwanese version of the Neurological Disorders Depression Inventory for Epilepsy (Tw-NDDI-E)

The NDDI-E is a six-item self-report measure designed to screen for depression in PWE over the past 2 weeks. Each item is rated on a scale of 0 (*never*) to 3 (*always or often*). It is accurate and reliable and can differentiate depressive symptoms from medication side effects, as well as cognitive deficits related to epilepsy (61).

The Tw-NDDI-E (54) is written in traditional Chinese, the official written language of Hong Kong (47); it exhibits good sensitivity (85%), specificity (87.64%), and positive predictive value (60.7%) (54). Although the test-retest reliability is not mentioned, the instrument exhibits excellent internal consistency (Cronbach's alpha = 0.90) and good concurrent validity with the Beck Depression Inventory-II (*r* = 0.825, *p* < 0.0001) (54). An optimal cut-off point of 15 was found (54).

#### 2.3.3 The Chinese version of the Generalized Anxiety Disorder 7-item scale (GAD-7)

The GAD-7 is a 7-item self-report instrument designed to identify probable GAD and measure its severity (62) over the past 2 weeks. Items are rated from 0 (*not at all*) to 3 (*nearly every day*). Scores of 5, 10, and 15 out of 21 serve as cut-off points for mild, moderate, and severe anxiety, respectively.

The GAD-7 has been validated in population-based samples (63, 64), a primary care sample (65), and among PWE (66), as well as in different languages (67, 68). The psychometric properties are good in some groups of PWE (66, 69). It was translated into traditional Chinese for Hong Kong by the MAPI Research Institute (55). Although it has not yet been formally validated in the general Hong Kong population, it has been validated in local adolescents aged 15 to 24 years (70) and is widely used by researchers and organizations in Hong Kong (71–76). Additionally, its simplified Chinese version for mainland China (77) and the traditional Chinese version for Taiwan (78) have been validated among PWE. Among Chinese PWE, the cut-off score of 6 yielded a sensitivity of 94% and a specificity of 91.4% (77).

#### 2.3.4 The Chinese version of the Depression Anxiety Stress Scales 21 (DASS-21)

The DASS-21 (79) contains three 7-item subscales to detect and distinguish between symptoms of depression (DASS-D), anxiety (DASS-A), and stress (DASS-S) over the past week. It is a self-report instrument, with answers rated on a 4-point Likert scale (ranging from '*does not apply to me'* to '*apply to me very much'*). Total scores for each subscale are multiplied by two to obtain final scores that range from 0 to 42. Good psychometric properties have been demonstrated (80, 81), and factor analysis has confirmed a 3-factor structure of depression, anxiety, and stress (81). The scale was validated in a Chinese sample, including Hong Kong Chinese, with a good fit from a 3-factor solution, and good construct validity was indicated by its moderate-to-high factor loadings (56). We, therefore, employed the DASS-21 to efficiently differentiate between symptoms of depression, anxiety, and stress.

#### 2.3.5 The Chinese version of the Liverpool Adverse Events Profile (LAEP)

The LAEP is a 19-item self-report measure developed in England to quantify the adverse effects of anti-seizure medications in the past month (82–84). Responses are rated on a 4-point Likert scale, ranging from 1 (*never a problem*) to 4 (*always a problem*). The LAEP has good internal consistency (Cronbach's alpha = 0.84–0.94) (85–88) and test-retest reliability (intraclass correlation coefficient = 0.81) (86).

The Chinese version of the LAEP (57) has 22 items, as the item “trouble with mouth/gums” was divided into “mouth problems” and “gum problems” (since the concepts of mouth and gums differ in Chinese). Two items—“weight loss” and “paresthesia”—were added upon expert consultation. It exhibits good internal consistency, test-retest reliability, a content validity index, and construct validity, as confirmed by factor analysis (57). Patients on polytherapy experienced more adverse effects and higher LAEP scores than those on monotherapy, indicating good concurrent validity (57).

### 2.4 Statistical analysis

We conducted statistical analysis using IBM SPSS Statistics Version 28.0. We established the significance level at *p* < 0.05.

We measured internal consistency using McDonald's omega (ω). We evaluated test-retest reliability through the Wilcoxon signed-rank test and Spearman's correlation. We set the retest interval at 2–4 weeks to minimize changes in clinical conditions or retention of previous responses.

We assessed construct validity using CFA, which was robust in indicating whether the data fit the hypothesized factor structures from previous studies (42–45), using the R package lavaan. We evaluated model fit based on the following criteria: comparative fit index (CFI) > 0.9, Tucker-Lewis index (TLI) > 0.9, root mean square error of approximation (RMSEA) < 0.08, and standardized root mean square residual (SRMR) < 0.08 (89). We examined convergent validity through the correlation between the TC-EASI and the GAD-7 and DASS-A, while we explored divergent validity through the correlation between the TC-EASI and the NDDI-E, DASS-D, and LAEP. We analyzed convergent and divergent validity using Spearman's rho correlation coefficients. A correlation coefficient ranging from 0.3 to 0.7 supports divergent validity, while a value above 0.7 indicates that the scales are equivalent in psychometric properties and probably measure the same phenomenon (90).

For the receiver operating characteristic (ROC) analysis of the TC-brEASI, we used the CB-SCID-I/P as the gold standard for diagnosing AD. To enhance comparability with other studies using DSM-5 criteria, participants diagnosed solely with OCD or PTSD were reclassified as not having an AD in the ROC analysis. Accordingly, we calculated the sensitivity, specificity, AUC, and their respective 95% confidence intervals. We determined the optimal cut-off point using the closest-to-(0,1) criterion (91). We compared the ROC between the TC-brEASI and GAD-7 in detecting AD and non-GAD anxiety disorders.

## 3 Results

### 3.1 Characteristics of the participants

Tables 2, 3 show the participants' characteristics. The median age was 41 years (interquartile range [IQR] = 31–57), and women accounted for 55.2% of the sample.

**Table 2:** Characteristics of the participants.

**Characteristics**	***n* (%)**	**Median (IQR)**
Age		41 (31–57)
Sex		
Male	91 (44.8%)	
Female	112 (55.2%)	
Education level		
Primary or below	23 (11.3%)	
Secondary	112 (55.2%)	
Tertiary or above	68 (33.5%)	
Employment status		
Employed	117 (57.6%)	
Housewife	31 (15.3%)	
Student	5 (2.5%)	
Unemployed	50 (24.6%)	
Marital status		
Single	91 (44.8%)	
Married	91 (44.8%)	
Divorced	11 (5.4%)	
Widowed	10 (4.9%)	
Age of onset		20 (13–34)
Duration of illness		16 (7–26)
Epilepsy type		
Generalized	68 (33.5%)	
Focal	135 (66.5%)	
Focus (for focal epilepsy)		
Temporal	96 (71.1%)	
Extratemporal	39 (28.9%)	
Seizure frequency (in the past year)		1 (0–10)
Duration of remission (months)		9 (0–39)
Drug resistant epilepsy	14 (6.9%)	
Resolved epilepsy	1 (0.5%)	
Anti-seizure medications	192 (94.6%)	
Number of anti-seizure medications		1 (1–2)
Antidepressants	20 (9.9%)	
Benzodiazepines	30 (14.8%)	
Defined daily dose (*N* = 30)		5 (2.69–10)
Vagal nerve stimulation	0 (0%)	

N = 203. IQR, interquartile range.

**Table 3:** Prevalence of current anxiety disorders in the sample.

**Anxiety disorder**	***n* (%)**
Any anxiety disorders	40 (19.7%)
Panic disorder	15 (7.4%)
Without agoraphobia	8 (3.9%)
With agoraphobia	7 (3.4%)
Agoraphobia without panic disorder	12 (5.9%)
Obsessive-compulsive disorder	10 (4.9%)
Social phobia	8 (3.9%)
Generalized anxiety disorder	8 (3.9%)
Specific phobia	2 (1%)
Post-traumatic stress disorder	2 (1%)
>1 anxiety disorders	12 (5.9%)

N = 203.

The median age of onset of epilepsy was 20 years (IQR = 13–34). The majority (66.5%) had focal epilepsy. Among those with focal epilepsy, 71.1% of them had a temporal focus. The median seizure frequency over the year was once. The median duration of seizure remission was 9 months (IQR = 0-39). A total of 7% of participants had drug-resistant epilepsy.

For treatment, 192 participants (94.6%) were on anti-seizure medications, with valproic acid being the most common (*n* = 75, 37%). A total of 20 (9.9%) participants were on antidepressants, with selective serotonin reuptake inhibitors as the most common type (*n* = 13, 6.4%). Moreover, 30 participants (14.8%) were on benzodiazepines.

The prevalence of current AD diagnosed by CB-SCID-I/P was 19.7% (95% CI = 14.2–25.2%). Panic disorder was the most prevalent individual AD (7.4%). A total of 12 individuals (5.9%) had more than one AD. The remaining 163 participants did not have a current AD.

### 3.2 TC-EASI scores

The median TC-EASI total score was 11 (IQR = 4–22). For the subscales, the median scores for typical anxiety symptoms, epilepsy-specific anxiety symptoms, and the TC-brEASI were 6 (IQR = 2–11), 6 (IQR = 2–12), and 5 (IQR = 2–9), respectively.

### 3.3 Psychometric results

#### 3.3.1 Internal consistency

The McDonald's omega for the TC-EASI total score was 0.95, demonstrating excellent internal consistency. The internal consistency of the subscales was also rated as good to excellent, with omega coefficients of 0.91 for typical anxiety symptoms, 0.91 for epilepsy-specific anxiety symptoms, and 0.89 for TC-brEASI.

#### 3.3.2 Test-retest reliability

A total of 25 participants completed the retest. The median number of days between the first and second administrations of TC-EASI was 17 (range: 14–24). As shown in Table 4, we found satisfactory test-retest reliability for the TC-EASI (*r* = 0.973, *p* < 0.001) and its subscales (*r* = 0.960-0.969, all *p* < 0.001). The Wilcoxon signed-rank test revealed no significant difference between test-retest scores.

**Table 4:** Test-retest reliability of the TC-EASI total and subscale scores.

**TC-EASI**	**Wilcoxon signed-rank test**	**Spearman's correlation test**	
	***p*** **-value**	**Speakman's rho**	***p*** **-value**
Total score	0.585	0.973	< 0.001
Typical anxiety symptoms	0.247	0.960	< 0.001
Epilepsy-specific anxiety symptoms	0.581	0.963	< 0.001
TC-brEASI	0.716	0.969	< 0.001

N = 25. TC-EASI, Traditional Chinese version of the Epilepsy Anxiety Survey Instrument. Typical anxiety symptoms comprised items 1, 2, 3, 4, 6, 10, 14, 15, and 17. Epilepsy-specific anxiety symptoms consisted of items 5, 7, 8, 9, 11, 12, 13, 16, and 18. TC-brEASI, Traditional Chinese version of the Brief Epilepsy Anxiety Survey Instrument.

#### 3.3.3 Construct validity

**Figure 2:**
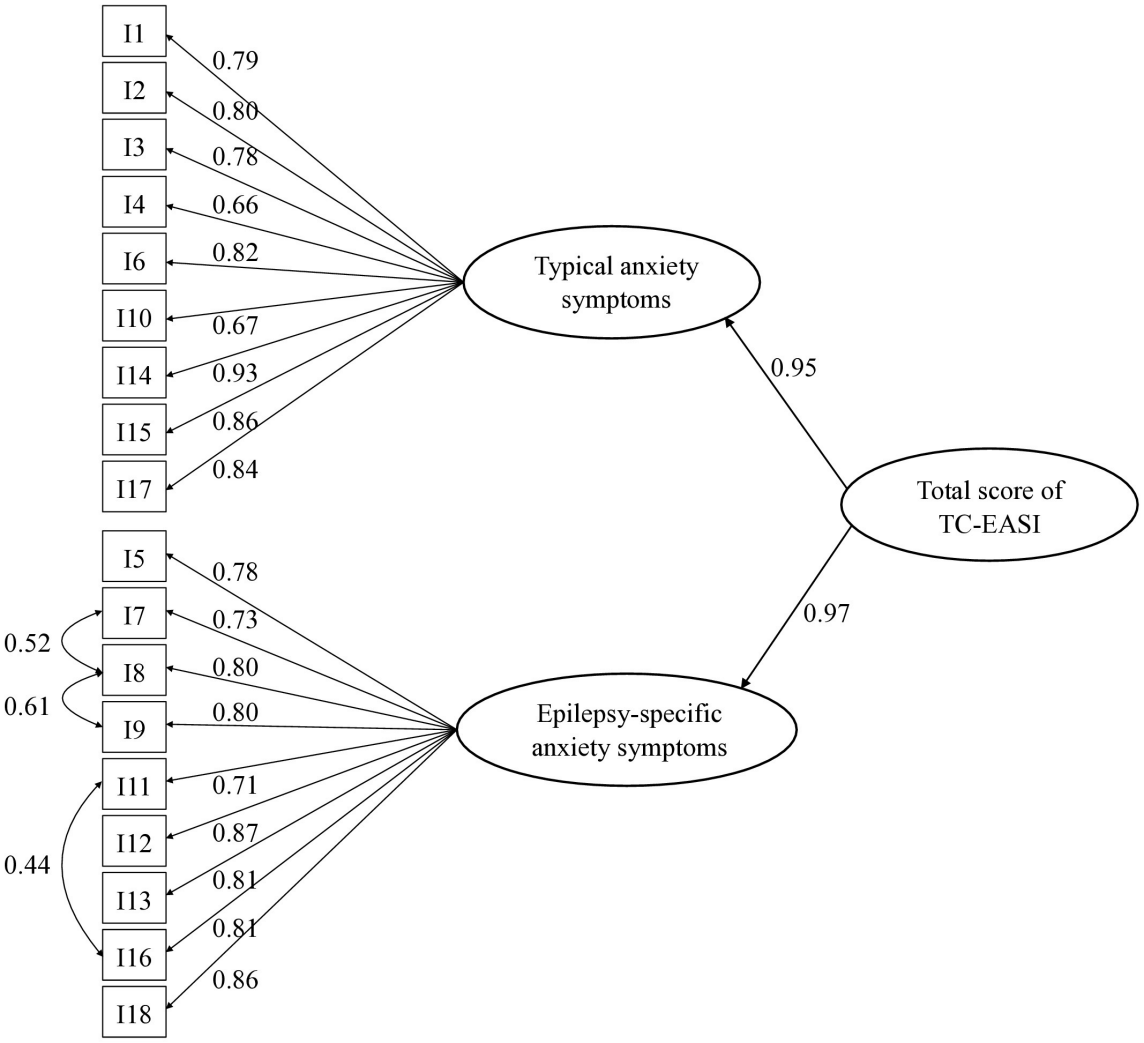
Two-factor CFA model of the TC-EASI. *N* = 203. CFA, confirmatory factor analysis; TC-EASI, Traditional Chinese version of the Epilepsy Anxiety Survey Instrument. Rectangles represent observed variables (the 18 items in the TC-EASI). Ellipses represent first- and second-order latent variables. The values next to the arrows represent standardized factor loadings.

We tested the one-factor, two-factor, three-factor, and four-factor models using CFA. Considering similarly worded item pairs, we examined the correlation of error terms. The two-factor model provided a good fit with our data, with fit indices shown in Table 5. Figure 2 depicts the path diagram of the two-factor model.

**Table 5:** Fit Indices for models of the TC-EASI using confirmatory factor analysis.

**Model**	**χ2**	**df**	**CFI**	**TLI**	**RMSEA (90% CI)**	**SRMR**
One-factor	451.109	135	0.949	0.942	0.108 (0.097–0.119)	0.076
Two-factor	415.653	134	0.955	0.948	0.102 (0.091–0.113)	0.069
Two-factor (error terms correlated)	321.376	131	0.969	0.964	0.085 (0.073–0.097)	0.060

N = 203. TC-EASI, Traditional Chinese version of the Epilepsy Anxiety Survey Instrument. The goodness-of-fit was evaluated based on the following criteria: comparative fit index (CFI) > 0.9; Tucker-Lewis index (TLI) > 0.9; root mean square error of approximation (RMSEA) < 0.08; and standard root mean square residual (SRMR) < 0.08.

#### 3.3.4 Convergent and divergent validity

From Table 6, the TC-EASI and TC-brEASI showed relatively strong correlations with the GAD-7 and DASS-A, along with weaker correlations with the DASS-D, NDDI-E, and LAEP.

**Table 6:** Convergent and divergent validity of the TC-EASI.

**Measure**	**TC-EASI**	
	**Total score**	**TC-brEASI**
	**Spearman's rho, all** ***p***<**0.001**	
GAD-7	0.76	0.78
DASS-21		
DASS-A	0.73	0.72
DASS-D	0.66	0.65
NDDI-E	0.67	0.65
LAEP	0.52	0.49

N = 203. TC-EASI, Traditional Chinese version of the Epilepsy Anxiety Survey Instrument. TC-brEASI, Traditional Chinese version of the Brief Epilepsy Anxiety Survey Instrument. DASS-21, Chinese version of the Depression Anxiety Stress Scales 21 (which includes the DASS-A, the anxiety subscale, and the DASS-D, the depression subscale). GAD-7, Chinese version of the Generalized Anxiety Disorder 7-item scale. LAEP, Chinese version of the Liverpool Adverse Events Profile. NDDI-E, Taiwanese version of the Neurological Disorders Depression Inventory for Epilepsy.

### 3.4 Cut-off score of the TC-brEASI

To enhance comparability with studies using DSM-5 criteria, we reclassified three participants with only OCD or PTSD as not having an AD. We found a cut-off score of 9 for the TC-brEASI based on the closest-to-(0,1) criterion, achieving a sensitivity of 89.2% (95% CI = 79.2–99.2%), a specificity of 82.5% (95% CI = 76.8–88.3%), and an AUC of 0.925 (95% CI = 0.887–0.964).

**Figure 3:**
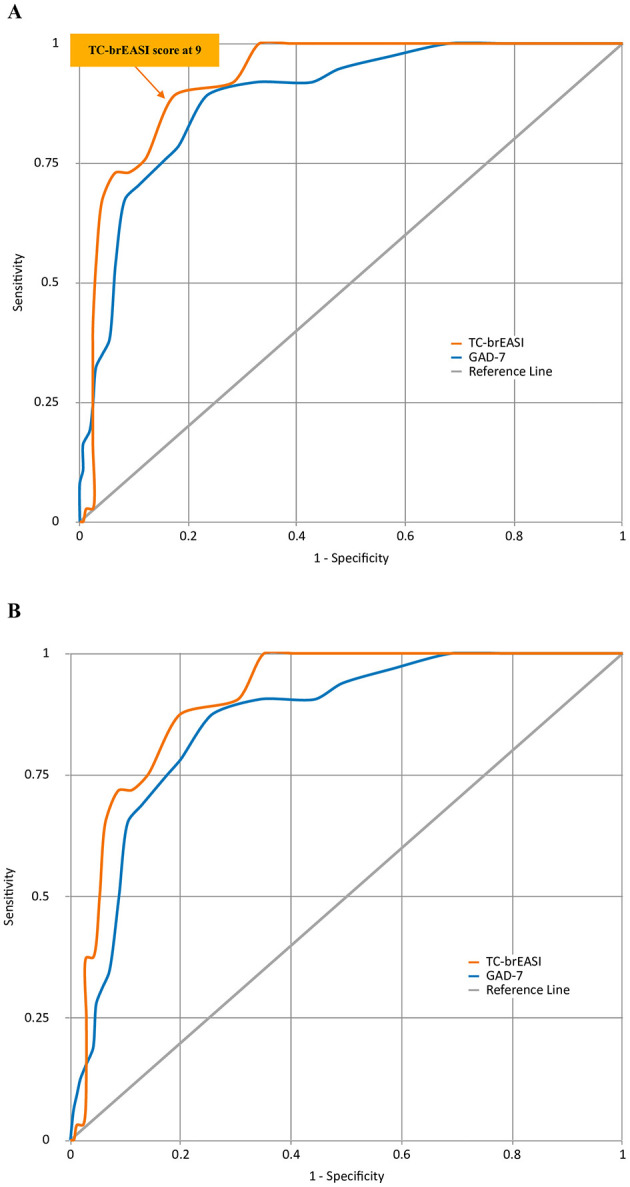
Comparison of ROC curves of the TC-brEASI and GAD-7 on anxiety disorders **(A)** and non-GAD anxiety disorders **(B)**. ROC, receiver operating characteristics; TC-brEASI, Traditional Chinese version of the Brief Epilepsy Anxiety Survey Instrument; GAD-7, Chinese version of the Generalized Anxiety Disorder 7-item scale; GAD, generalized anxiety disorder.

The pairwise comparison of the ROC of the TC-brEASI and GAD-7 regarding AD and non-GAD anxiety disorders (Table 7, Figures 3A, B) showed that the TC-brEASI achieved an excellent AUC of 0.907 for detecting non-GAD anxiety disorders as well. On the other hand, the GAD-7 showed a numerically lower AUC. In our sample, 30.5% (95% CI = 24.2–36.9%) of participants scored above the TC-brEASI cut-off point for AD, while 35.5% (95% CI = 28.9–42%) scored above the GAD-7 cut-off point for AD.

**Table 7:** Receiver operating characteristics of TC-brEASI and GAD-7 for anxiety disorders and GAD.

**Measure**	**TC-brEASI**	**GAD-7**	**TC-brEASI**	**GAD-7**
**Intended disorders**	**Anxiety disorders**	**Anxiety disorders**	**Non-GAD anxiety disorders**	**Non-GAD anxiety disorders**
Cut-off score	≥ 9	≥ 7	≥ 9	≥ 7
Sensitivity (95% CI)	89.2% (79.2–99.2%)	89.2% (79.2–99.2%)	87.5% (76.0–99.0%)	87.5% (76.0–99.0%)
Specificity (95% CI)	82.5% (76.8–88.3%)	76.5% (70.1–83.0%)	80.1% (74.1–86.1%)	74.3% (67.7–80.8%)
PPV (95% CI)	53.2% (40.8–65.6%)	45.8% (34.3–57.3%)	45.2% (32.8–57.5%)	38.9% (27.6–50.1%)
NPV (95% CI)	97.1% (94.4–99.9%)	96.9% (94.0–99.9%)	97.2% (94.4–99.9%)	96.9% (94.0–99.9%)
AUC (95% CI)	0.925 (0.887–0.964)	0.886 (0.831–0.941)	0.907 (0.862–0.951)	0.861 (0.799–0.93)

N = 203. TC-brEASI, Traditional Chinese version of the Brief Epilepsy Anxiety Survey Instrument; CI, confidence interval; GAD, generalized anxiety disorder; GAD-7, Chinese version of the Generalized Anxiety Disorder 7-item scale; PPV, positive predictive value; NPV, negative predictive value; AUC, area under the curve.

### 3.5 Subgroup analysis for anxiety disorders (excluding OCD and PTSD)

The TC-EASI total and subscale scores did not differ significantly after excluding participants with OCD and PTSD from those diagnosed with AD using the CB-SCID-I/P, as shown in Table 8.

**Table 8:** Subgroup analysis for anxiety disorders (excluding OCD and PTSD).

**TC-EASI, median (IQR)**	**CB-SCID-I/P diagnoses**		***p*-value**
	**Anxiety disorders**	**Anxiety disorders (excluding OCD and PTSD)**	
Total	31 (26–37.75)	31 (24.25–39)	0.822
Typical anxiety	14 (9–16)	14.5 (9.25–16)	0.900
Epilepsy-specific anxiety	17 (13–20)	17.5 (13–20.75)	0.731
TC-brEASI	13.5 (9.25–16)	14 (9.25–17.5)	0.846

N = 203. Data were analyzed using the Mann-Whitney U test. TC-brEASI, Traditional Chinese version of the Brief Epilepsy Anxiety Survey Instrument; TC-EASI, Traditional Chinese version of the Epilepsy Anxiety Survey Instrument; IQR, interquartile range; OCD, obsessive-compulsive disorder; PTSD, post-traumatic stress disorder; CB-SCID-I/P, the Chinese Bilingual version of the Structured Clinical Interview for DSM-IV-TR Axis I Disorder, Research Version, Patient Edition.

## 4 Discussion

### 4.1 Psychometric properties of the TC-EASI

A total of 203 participants were recruited, resulting in a satisfactory participation rate of 82.9%. The participants and non-participants did not differ significantly in age or sex.

The internal consistency was good for the TC-EASI (omega = 0.95) and TC-brEASI (omega = 0.89). These results were comparable to the Russian study (omega = 0.93 for the EASI and 0.88 for the brEASI). Good internal consistency was also found in the original study (alpha = 0.94 for the EASI and 0.91 for the brEASI), in Germany (alpha = 0.94 for the EASI and 0.86 for the brEASI), in France (alpha = 0.90 for the EASI), and in Western China (alpha = 0.94 for the EASI and 0.873 for the brEASI). Test-retest reliability was also excellent for both scales.

CFA revealed a good fit in the two-factor model. The model seemed related to the dimensions of “typical anxiety” and “epilepsy-specific anxiety.” This was corroborated by the original and French studies. Notably, in the French study, item 14 (“I worried about situations where I might make a fool of myself”) was related to epilepsy-specific anxiety instead of the typical anxiety via principal component analysis. In the original study, item 14 fell under typical anxiety; it made clinical sense to represent a spectrum of social anxiety that is not just limited to situations related to seizures. In our study, we found a standardized factor loading of 0.93 for item 14, showing a high correlation with typical anxiety. Otherwise, the items of the two dimensions in the original and French versions were the same. A three-factor model was reported in the Russian study, appearing to consist of “internal general anxiety symptoms,” “external general anxiety symptoms,” and “epilepsy-specific anxiety.” The factor structure was not reported in the German or Western Chinese studies (46).

The convergent validity of the TC-EASI was demonstrated by its strong correlation with the total scores of the GAD-7 (*r* = 0.76 for the TC-EASI and 0.78 for the TC-brEASI) and the DASS-A (*r* = 0.73 for the TC-EASI and 0.72 for the TC-brEASI). This aligns with findings using GAD-7 in the original, French, German, and Russian studies (*r* = 0.70–0.82 for the EASI and 0.79–0.89 for the brEASI) (42–45) as well as the DASS-A in the original study (*r* = 0.71 for the EASI and 0.69 for the brEASI) (42).

Divergent validity was supported by weaker correlations with the NDDI-E (*r* = 0.67 for the TC-EASI and 0.65 for the TC-brEASI), DASS-D (*r* = 0.66 for the TC-EASI and 0.65 for the TC-brEASI), and LAEP (*r* = 0.52 for the TC-EASI and 0.49 for the TC-brEASI). Measures of depression (NDDI-E and DASS-D) still exhibited moderate correlations with the TC-EASI and TC-brEASI, echoing the findings of all the above cross-cultural EASI validation studies, which reported similar correlations with the NDDI-E (*r* = 0.58–0.70 for the EASI and *r* = 0.69–0.80 for the brEASI) (42–45). GAD-7 validation studies among PWE in France and South Korea have also demonstrated a moderate to strong correlation (*r* = 0.66–0.77) with the NDDI-E (66, 92). This may have been attributed to the overlap and comorbidity between depression and anxiety (19, 93, 94).

Overall, our evidence suggests that the TC-EASI and TC-brEASI have good psychometric properties.

#### 4.1.1 Determining the cut-off score

We determined a cut-off score of ≥ 9 using the closest-to-(0,1) criterion, with which the TC-brEASI performed robustly, achieving an AUC of 0.925, a sensitivity of 89.2%, and a specificity of 82.5%. It demonstrated the highest AUC among existing cross-cultural EASI validation studies. This strong performance is vital as a screening tool to effectively discriminate between PWE with and without AD. It detected 30.5% of our sample as having probable AD, while the GAD-7 indicated 35.5%, and the CB-SCID-I/P-determined prevalence of current AD was 19.7%.

The cut-off score of our TC-brEASI differed from those reported in the original (cut-off score ≥7, AUC = 0.891) (42), French (cut-off score ≥8, AUC = 0.757) (43), German (cut-off score ≥6, AUC = 0.90) (44), Russian (cut-off score ≥8, AUC = 0.92) (45), and Western Chinese (cut-off score ≥8, AUC of 0.883) (46) studies. However, applying the original cut-off score of ≥7 to our sample would result in improved sensitivity (100%) but at a considerable expense of specificity (68.1%).

A specific comparison with the Western Chinese study reveals a one-point difference in the optimal cut-off score, which can be explained from both clinical and methodological perspectives. Clinically, the prevalence of anxiety disorders in our sample (19.7%) was lower than that in the Western Chinese sample (23.6%). The severity of anxiety in our sample was also lower, as indicated by consistently lower mean scores on the EASI, brEASI, and GAD-7 scores (14.8 vs. 17.6, 6.4 vs. 7.27, and 5.7 vs. 6.5 in our study and the Western Chinese study, respectively). Therefore, both the prevalence and the severity of anxiety were lower in our sample, potentially resulting in a higher optimal cut-off score to maintain both sensitivity and specificity. Methodologically, there were substantial differences in sample sizes (203 in our study vs. 110 in the Western Chinese study) and sampling periods (5 months vs. 2 months). A larger sample size and longer sampling period can introduce greater heterogeneity and representativeness, which may affect the distribution of scores and, consequently, the determination of the optimal cut-off score.

### 4.2 Prevalence of current anxiety disorders in PWE

We found that the current prevalence of AD in PWE is 19.7%, compared to the worldwide pooled prevalence of 20.2% in a meta-analysis (32) and 25.6% in a systematic review (19). It is likely higher than in our general local population, as indicated by a 2015 study, although different detection methods and recall periods were used (38). There are relatively few recent local studies on the prevalence of AD among adult PWE (20, 21).

Among individuals with AD in the PWE population, panic disorder was the most prevalent (7.4%), followed by agoraphobia without panic disorder (5.9%). However, if agoraphobia and panic disorder were considered separate diagnoses, as defined in the DSM-5, agoraphobia would be the most prevalent AD (9.3%). We found that 5.9% of PWE suffered from more than one current AD, compared to 6.1% to 13.3% reported in the Australian, German, and Russian studies (44, 45, 95).

The prevalence of current GAD (3.9%) in our study was relatively low compared to the pooled prevalence of 10.2% in a meta-analysis on PWE (32) and 11.1% from a systematic review (19). In contrast, the prevalences of panic disorder (7.4%) and agoraphobia (9.3%) were higher than those reported in the meta-analysis (2.6% and 2.8%, respectively) (32) and the systematic review (7.0% for panic disorder and not available for agoraphobia) (19). Current literature indicates that the prevalences of both GAD and panic disorder are known to vary significantly by country/region and income level (96, 97). The difference has been attributed to factors such as methodological differences, the reporting of mental illness, and diverse risk and protective factors (97). The prevalence of GAD found in our study was similar to that in local studies (4.1% and 4.2%) conducted in the general Hong Kong population (38, 98). Meanwhile, another possible explanation specific to GAD and panic disorder is the tendency in Asian populations to express and report anxiety through somatic symptoms rather than psychological symptoms (98–100), leading to a higher likelihood of meeting the criteria for panic disorder than for GAD. This is a well-known phenomenon and is cited as a limitation of the DSM-IV GAD criteria due to its psychological emphasis and overlap with depressive symptoms (101, 102). The Western Chinese validation study reported a similar prevalence of panic disorder (6.3%) but a significantly higher prevalence of GAD (20.0%) despite the differences in sampling period, gold standard diagnostic tool, rate of resistant epilepsy, and likely urban/rural composition. Therefore, this hypothesis may only partially, but not fully, explain the differences in AD prevalence. A final hypothesis is that the pattern of anxiety in Hong Kong PWE is more episodic and health-focused rather than chronic and generalized. However, limited literature explores the pattern and theme of anxiety in Hong Kong PWE, and it may be beneficial to research this further.

### 4.3 Clinical implications

This study introduced the first epilepsy-specific tools in traditional Chinese—the TC-EASI and TC-brEASI—for detecting and assessing AD among PWE in Hong Kong. Both measures are reliable and valid. With strong sensitivity and specificity, the TC-brEASI is an efficient tool to use alongside the NDDI-E for screening probable depression and AD among PWE in busy neurology clinics.

The longer TC-EASI, apart from comprehensively informing about the nature and severity of anxiety, can detect epilepsy-specific anxiety (such as anticipatory anxiety of seizures). It also enables clinicians to understand a patient's perspective and life difficulties related to epilepsy, allowing for tailored suggestions. For the patient, the questionnaire also provides an opportunity to develop a deeper understanding of their psychological needs.

Epidemiologically, our study indicates that PWE urgently needs mental health services, given the high prevalence of current AD (19.7%). This highlights the necessity for psychiatric screening using our epilepsy-specific instrument, which would hopefully mitigate the “diagnostic gap” of AD in PWE and its impact.

By putting insights into practice, we advocate for enhanced mental health coverage in epilepsy care. To begin with, a yearly screening service for common mental disorders (TC-brEASI and NDDI-E) could be integrated into the epilepsy clinic. This would represent a response to global advocates of psychiatric screening among PWE (41, 103) and help overcome barriers to screening practices (31). This method has been used in the United States to improve detection and treatment rates (104). Next, we suggest that where feasible, trained clinical team members (such as epilepsy nurse specialists or other qualified professionals) can be involved in the follow-up of PWE who screen as positive. They can (a) administer the longer TC-EASI to understand more about the nature and severity of the patient's anxiety, (b) identify any risk factors for developing psychiatric disorders, (c) address any epilepsy-specific anxious cognitions, and (d) empower patients to cope with epilepsy-specific life difficulties and regulate their emotions. Any significant findings can be reported to the epileptologist for medical advice and referral to specialized mental health services where appropriate. Looking ahead, these measures can improve the mental health of PWE, promote proper healthcare service usage, and reduce the personal and societal impact of epilepsy.

Our findings, therefore, have both epidemiological and clinical implications, including diagnostic, therapeutic, and preventive applications.

### 4.4 Strengths of this study

Our study has several strengths. It is the first to translate and validate a traditional Chinese version of the EASI to screen and assess AD among PWE. Participants were recruited from an epilepsy clinic dedicated to PWE, which serves approximately 15% of Hong Kong's population (105).

The satisfactory participation rate was 82.9%, exceeding the required sample size. We adopted a stringent methodology in the process of validating the TC-EASI, with major modules of the CB-SCID-I/P administered to all participants to diagnose any mental illnesses. The resulting TC-brEASI achieved an excellent AUC, indicating strong discriminative power between true positives and false negatives.

### 4.5 Limitations of this study

Our study also has several limitations. First, due to its cross-sectional nature, we were unable to determine sensitivity to changes in the TC-EASI or establish a causative relationship among the variables. Second, recruiting through convenience sampling over approximately 5 months may have biased the sample toward individuals with shorter follow-up intervals and, consequently, less clinically stable epilepsy. Participants were recruited exclusively from a specialized clinic within one of Hong Kong's seven public healthcare clusters. This may have led to bias in the sample due to differences in epilepsy severity compared with those under the care of private doctors and psychiatrists or those not receiving any medical care. Nonetheless, in Hong Kong, the majority of PWEs are managed in the public sector (106). Therefore, the impact on the generalizability of our findings is likely limited.

On the other hand, as socioeconomic inequalities in Hong Kong have been known to be associated with anxiety and self-perceived health (107, 108), replicating our single-center findings in a more socioeconomically diverse sample will enhance generalizability and provide a more comprehensive understanding. Third, patients with PNES and intellectual disabilities (who were excluded from the study) cannot benefit from our instrument and findings. Fourth, some participants may have undiagnosed PNES in the epilepsy clinic (109). Fifth, the divergent validity of the scales was not easily demonstrated, especially between depression and anxiety measures. Aside from the use of Spearman's rho, no additional conventional statistical methods (such as the Heterotrait-Monotrait ratio of correlations) were used. Finally, we employed the CB-SCID-I/P based on DSM-IV-TR diagnoses since a validated traditional Chinese version of the SCID-5 was not available at the time of the study. The most notable change in the DSM-5 is that OCD and PTSD are excluded from AD. Nonetheless, we found no significant differences in the TC-EASI scores of participants with AD after excluding those with OCD and PTSD.

### 4.6 Directions for future research

This study has paved the way for local research on epilepsy-specific anxiety. Future studies should explore the presence of epilepsy-specific anxiety as a distinct nosological entity and clarify the threshold between normal and disordered anxiety in the local context. Additionally, a longitudinal study would be valuable for assessing sensitivity to change and establishing the predictive validity of the TC-EASI, supporting its use as a monitoring and prognostic tool. It can also be used to examine causal relationships, such as the effect of medications on mood and seizures. Finally, further research would benefit from the availability of a traditional Chinese version of the SCID-5 in the future.

### 4.7 Other information

We received no specific grant from any funding agency in the public, commercial, or not-for-profit sectors. The study protocol is available from the corresponding author upon reasonable request. There are no missing data.

## 5 Conclusion

We developed the TC-EASI and TC-brEASI as reliable and valid self-report tools for assessing and screening AD among PWE in Hong Kong. These tools provide valuable insight into the nature and severity of both typical and epilepsy-specific anxiety. The TC-brEASI can be used alongside the NDDI-E to screen for anxiety and depression in clinical settings, with the aim of improving mental health coverage and better addressing the service needs of PWE in Hong Kong.

## Data Availability

The raw data supporting the conclusions of this article will be made available by the authors, without undue reservation.
