# Supplementary material for: Validating the Traditional Chinese version of the Epilepsy Anxiety Survey Instrument (EASI) in Hong Kong
Source: Front Neurol. 2025 Jul 8;16:1604317. doi: 10.3389/fneur.2025.1604317 (PMC12279500; doi:10.3389/fneur.2025.1604317)
Supplement: Supplementary file 1 [file Table_1.docx]

**Traditional Chinese Version of Epilepsy Anxiety Survey Instrument (TC-EASI)**

癲癇焦慮量表

**EASI-18**

在過去的兩星期，你有多經常被以下任何問題所困擾？（請圈出適當的數字）

|  | 完全沒有 | 偶有幾天 | 過半日子 | 幾乎每天 |
| --- | --- | --- | --- | --- |
| 1. 我一開始擔憂便無法制止或控制 | 0 | 1 | 2 | 3 |
| 1. 我發現難以放鬆自己 | 0 | 1 | 2 | 3 |
| 1. 擔憂的想法閃過我的腦海 | 0 | 1 | 2 | 3 |
| 1. 我突然感到驚慌，但這與我腦癇發作無關 | 0 | 1 | 2 | 3 |
| 1. 我發現難以制止對腦癇發作的擔憂 | 0 | 1 | 2 | 3 |
| 1. 害怕和焦慮令我無法做重要的事情 | 0 | 1 | 2 | 3 |
| 1. 如果我害怕某事情會令腦癇發作，我會完全避開它 | 0 | 1 | 2 | 3 |
| 1. 我避開不去如果腦癇發作便難以求助的地方 | 0 | 1 | 2 | 3 |
| 1. 我避開不去別人可以看到我腦癇發作的地方 | 0 | 1 | 2 | 3 |
| 1. 我在無預期下感到極度恐懼，但這與我腦癇發作無關 | 0 | 1 | 2 | 3 |
| 1. 我時常想我有可能腦癇發作 | 0 | 1 | 2 | 3 |
| 1. 我擔憂別人對我的看法 | 0 | 1 | 2 | 3 |
| 1. 我擔憂腦癇對四周各人的影響 | 0 | 1 | 2 | 3 |
| 1. 我擔憂某些場合可能令自己出醜 | 0 | 1 | 2 | 3 |
| 1. 我發現難以自我享受，因為我無法擺脫擔憂 | 0 | 1 | 2 | 3 |
| 1. 當我留意到與腦癇有關的徵狀出現，便感到驚怕 | 0 | 1 | 2 | 3 |
| 1. 普遍情況下，我往往會預料或預測最壞的結果出現 | 0 | 1 | 2 | 3 |
| 1. 我擔憂自己會否做出某事情導致腦癇發作 | 0 | 1 | 2 | 3 |

| 總分： |  |
| --- | --- |

**brEASI**

在過去的兩星期，你有多經常被以下任何問題所困擾？（請圈出適當的數字）

|  | 完全沒有 | 偶有幾天 | 過半日子 | 幾乎每天 |
| --- | --- | --- | --- | --- |
| 1. 我一開始擔憂便無法制止或控制 | 0 | 1 | 2 | 3 |
| 1. 我發現難以放鬆自己 | 0 | 1 | 2 | 3 |
| 1. 擔憂的想法閃過我的腦海 | 0 | 1 | 2 | 3 |
| 1. 我突然感到驚慌，但這與我腦癇發作無關 | 0 | 1 | 2 | 3 |
| 1. 害怕和焦慮令我無法做重要的事情 | 0 | 1 | 2 | 3 |
| 1. 我避開不去如果腦癇發作便難以求助的地方 | 0 | 1 | 2 | 3 |
| 1. 我擔憂某些場合可能令自己出醜 | 0 | 1 | 2 | 3 |
| 1. 我發現難以自我享受，因為我無法擺脫擔憂 | 0 | 1 | 2 | 3 |

| 總分： |  |
| --- | --- |

計分指引

**TC-EASI:** 分數由 0 至 54 不等，分數越高表示焦慮越嚴重。

第 1、2、3、4、6、10、14、15 和 17 項反映較典型的焦慮症狀；

第 5、7、8、9、11、12、13、16、18 項反映與腦癇症有關的焦慮症狀。

**TC-brEASI:** 分數由 0 至 24 不等，分數等於或高於 **9** 分表示可能患有焦慮症。
